# Durable T-cellular and humoral responses in SARS-CoV-2 hospitalized and community patients

**DOI:** 10.1371/journal.pone.0261979

**Published:** 2022-02-22

**Authors:** Kristin G.-I. Mohn, Geir Bredholt, Fan Zhou, Anders Madsen, Therese B. Onyango, Elisabeth B. Fjelltveit, Sarah L. Jalloh, Karl A. Brokstad, Diego Cantoni, Martin Mayora-Neto, Nigel Temperton, Nina Langeland, Rebecca J. Cox, on behalf of Bergen COVID-19 research group

**Affiliations:** 1 Influenza Centre, Bergen, Norway; 2 Department of Medicine, Bergen, Norway; 3 Department of Microbiology, Bergen, Norway; 4 Department of Clinical Science, Broegelmann Research Laboratory, University of Bergen, Bergen, Norway; 5 Department of Safety, Chemistry and Biomedical Laboratory Sciences, Western Norway University of Applied Sciences, Bergen, Norway; 6 Viral Pseudotype Unit, Medway School of Pharmacy, University of Kent, Chatham, United Kingdom; 7 National Advisory Unit for Tropical Infectious Diseases, Bergen, Norway; University of Pittsburgh, UNITED STATES

## Abstract

**Background:**

Neutralizing antibodies are important for protection against the pandemic SARS-CoV-2 virus, and long-term memory responses determine the risk of re-infection or boosting after vaccination. T-cellular responses are considered important for partial protection against novel variants of concern.

**Methods:**

A prospective cohort of hospitalized (n = 14) and community (n = 38) patients with rt-PCR confirmed SARS-CoV-2 infection were recruited. Blood samples and clinical data were collected when diagnosed and at 6 months. Serum samples were analyzed for SARS-CoV-2-spike specific antibodies using ELISA (IgG, IgA, IgM), pseudotype neutralization and microneutralization assays. Peripheral blood mononuclear cells were investigated for virus-specific T-cell responses in the interferon-γ and interleukin-2 fluorescent-linked immunosorbent spot (FluroSpot) assay.

**Results:**

We found durable SARS-CoV-2 spike- and internal protein specific T-cellular responses in patients with persistent antibodies at 6 months. Significantly higher IL-2 and IFN-γ secreting T-cell responses as well as SARS-CoV-2 specific IgG and neutralizing antibodies were detected in hospitalized compared to community patients. The immune response was impacted by age, gender, comorbidity and severity of illness, reflecting clinical observations.

**Conclusions:**

SARS-CoV-2 specific T-cellular and antibody responses persisted for 6 months post confirmed infection. In previously infected patients, re-exposure or vaccination will boost long-term immunity, possibly providing protection against re-infection with variant viruses.

## Introduction

The novel severe acute respiratory coronavirus 2 (SARS-CoV-2) was first reported in humans in Wuhan, China, causing severe viral pneumonia and death. The virus has subsequentially spread globally, causing the most devastating pandemic since the Spanish influenza A/H1N1 in 1918. The clinical characteristics of SARS-CoV-2 disease have been well described [1, 2]. SARS-CoV-2 virus is primarily a trigger for an immunological illness, which affects several organ systems. The severity of illness is dependent on age and comorbidity and related to the individual’s primary immunological response. The quality of the long-term immune response determines the risk of re-infection. Detailed immunological knowledge, however, is limited and primarily focused on antibody responses. Early clinical observations of gender differences during acute infection found that males had a higher risk of severe disease and mortality [3, 4]. These findings have been supported by reports of immunological differences related to gender, such as less robust T-cell responses in males [5] and findings of sex differences in immune responses to vaccines and infection [6]. Most infected people seroconvert but reports of antibody waning and heterogeneity in antibody responses among infected people, have caused concern for the long-term protection after infection and particularly with the ongoing vaccination campaign [7, 8] The protective antibody level is unknown, and there is no agreed correlate of protection to date [9].

Community protection is the goal of SARS-CoV-2 mass vaccination. Similarly, protection from re-infection is dependent upon long-term memory elicited after primary infection. The immune response is essential and correlates with the severity of SARS-CoV-2 infection [10, 11]. Eighteen months has passed since the start of the pandemic and the global research conducted is unprecedented in speed and magnitude. Naturally, there is substantially less knowledge of durable immune responses after SARS-CoV-2 compared to acute immune responses.

T cells support antibody production by providing a prolonged B-cell response. However, the evidence of re-infection and short-lived immunity against the human coronaviruses (HCoV) has raised concern that immunity could be short lived [12]. With antibody titers waning over time, cellular immune responses, both B and T cells will be vital in limiting disease severity [13, 14]. Indeed, cellular protection has been confirmed in an animal challenge model [15]. Although recent studies find robust cellular immune responses post-infection, their longevity is unknown, however reports of more than six months and reports of persistent MBCs in the elderly despite reduction in neutralizing antibodies have been made [16]. Encouragingly, cellular responses after SARS in 2003 were found up to 6 years post-infection and are thought to last longer compared to antibody responses [17].

Here we report on durable SARS-CoV-2 specific antibody and T-cellular immune responses 6 months post-infection in rt-PCR (reverse transcription polymerase chain reaction) confirmed cases of varying disease severity (community and hospitalized patients) in a prospective cohort study.

## Methods

### Patients and study design

Patients were prospectively recruited during the first pandemic wave in Bergen, Norway (March- June 2020) from patients diagnosed at a centralized out-patient clinic (n = 86, mildly to moderately ill), and from hospitalized patients (n = 14 with moderate to severe disease needing oxygen or ICU treatment). Informed consent was obtained prior to recruitment (from the next of kin for patients in ICU) and follow-up blood samples collected two- and six-months post-infection [18, 19]. The study was approved by the Regional Committee for Medical and Health Research Ethics in Western Norway (#118664). An electronic case report form (eCRF) was used to collect relevant clinical and demographic data using Research Electronic Data Capture tools (REDCap, Vanderbilt, US) (Table 1). The eCRF contained information on gender, age, symptoms of COVID-19, rt-PCR test result, comorbidities and medication, treatment, and outcome.

**Table 1:** Patient demographics.

	Community patients	Hospitalized patients	
Total:	n = 38	n = 14	
Age (mean, range)	49 (19–80)	60 (45–75)	ns
Days since diagnose median (range)	188 (171–245)	182 (154–204)	ns
Female	17 (45%)	6 (43%)	ns
Male	21 (55%)	8 (57%)	ns
With any comorbidity (incl BMI >30 obesity grade 1)	14 (37%)	11(79%)	P 0.008
Known comorbidity (–BMI)	14 (37%)	9 (64%)	P = 0.077
Diabetes	2 (5%)	2 (14%)	ns
Hypertension	6 (16%)	6 (43%)	p = 0.04
Asthma	4 (11%)	1 (7%)	ns
Chronic lung disease (excluding asthma)	0 (0%)	2 (14%)	ns
Chronic heart disease	4 (11%)	5 (36%)	P = 0.05
Chronic renal disease	0 (0%)	1 (7%)	na
Chronic hepatic disease	0 (0%)	1 (7%)	na
Chronic neurological disease	0 (0%)	1(7%)	na
Cancer	1 (3%)	1 (7%)	na
BMI m^2^/kg (median)	24.6	27.1	p = 0.05

The demographics of the SARS-Cov-2 infected patients recruited during the first pandemic wave in March/April in Bergen, Norway. The community dwelling patients with symptoms, were recruited from the communal out-patient clinic, after rt-PCR confirmed SARS-CoV-2 infection (n = 38). The hospitalized patients were recruited from the pandemic wards at the Haukeland University Hospital or Haraldsplass Deaconal Hospital Bergen, Norway (n = 14). Clinical data was collected using an e-CRF (Red-Cap) and peripheral blood mononuclear cells (PBMCs) were separated and used in the T-cell assays and serum was used in the antibody assays.

### Serum and peripheral blood mononuclear cells (PBMC)

Blood samples were collected at two and six months after diagnosis and sera were stored at −80°C until used. PBMCs were isolated using Cell Preparation tubes (CPT, BD, UK), resuspended in RPMI-1640 supplemented with 10% fetal bovine serum, counted and diluted to appropriate concentration (2x10^6^ cells/ml) and used directly in T-cell FluroSpot assays.

### Virus, antigens, and peptides

The hCoV-19/Norway/Bergen-01/2020 (GISAID accession ID EPI_ISL_541970) virus was isolated in-house from an rt-PCR-confirmed patient in March 2020 and propagated in Vero cells before use in the microneutralization assay. In our local clinical isolate there are 2 amio acid differences in the spike protein: D614G and R682L compared to the Wuhan-Hu-1 strain. The SARS-CoV-2 (Wuhan-Hu-1 isolate) receptor binding domain (RBD) and spike proteins were produced in-house from constructs provided by Professor Florian Krammer [20].

Libraries of synthetic peptides (> 80% pure) covering the full length of the SARS-CoV-2 spike protein (S), nucleocapsid protein (N) and matrix protein of the USA-WA1/2020 strain were obtained from BEI Resources (VA, USA). The a.a. sequences of these proteins are identical to the respective proteins of the Wuhan-Hu-1 strain. The peptides were 17-mers, with 10 amino acid overlaps. The C-terminal peptides of each protein were either 12-mer (M) or 13-mer (S and N). The peptides were solubilized in anhydrous DMSO (≥ 99.9%), pooled and diluted in medium to a final DMSO concentration of < 0.5%. The peptides for the S protein were combined in two distinct pools, S1 (a.a.1-689) covering the main part of the S1 subunit and S2 (a.a.680-1273) covering the main part of the somewhat more conserved S2 subunit.

### Enzyme-linked immunosorbent assay (ELISA)

The spike protein ELISA was performed as previously described, but with some modifications [18, 20, 21]. Sera were serially diluted in a 5-fold manner from 1:100 and run in duplicate. The horseradish peroxidase (HRP)-labelled secondary antibodies directed against IgG (SouthernBiotech, Birmingham, AL, USA), IgA and IgM (Sigma-Aldrich, St. Lois, MO, USA) were detected with the chromogenic substrate 3,3´,5,5´-tetramethylbenzidine (TMB; BD Biosciences, San Jose, CA, USA). Optical density (OD) was measured at 450/620 nm using the Synergy H1 Hybrid Multi-Mode Reader with the Gen5 2.00 (version 2.00.18) software (BioTek Instruments Inc., Winooski, VT, USA). Endpoint titers were determined for IgG, IgA and IgM. Positive controls were serum from a hospitalized COVID-19 patient and CR3022 [22], whereas pooled pre-pandemic sera (n = 128) were used as a negative control [21]. Samples with no detectable antibodies were assigned an a titer of 50 for calculation purposes.

### Pseudotype neutralization (PN) assay

The PN assay was conducted with the pseudotype of the infecting virus D614G as described in [23]. Briefly, lentiviral pseudotypes were generated by transfecting HEK293T cells with plasmids encoding the SARS-CoV-2 Spike with D614G mutation, p8.91 Gag-pol and pCSFLW luciferase reporter. Cells were incubated for 48 hours prior to harvesting and filtering of the culture media through a 0.45μm cellulose acetate filter. Pseudotypes were titrated and quantified based on the relative luminescence units per ml (RLU/ml). For PN assays, sera were mixed with pseudotypes and serially diluted (from 1:40). HEK293T cells expressing ACE2 and TMPRSS2 were seeded at a density of 10.000 cells per well and plates were incubated for 48 hours prior to lysis using Bright-Glo (Promega) to measure reporter activity on a luminometer.

### Microneutralization (MN) assays

Paired sera were tested in the microneutralization (MN) assay, performed in a certified Biosafety Level-3 Laboratory using the live hCoV-19/Norway/Bergen-01/2020 (GISAID accession ID EPI_ISL_541970) virus as previously described [18, 21]. Briefly, serially diluted sera (from 1:20) and 100 tissue-culture infectious dose 50% (TCID_50_) virus were incubated for 1 hour at 37°C before 24-hour incubation at 37°C with Vero cells. The MN titer was calculated as the reciprocal of the serum dilution giving 50% inhibition of virus infectivity. Titers <20 were assigned a value of 10 for calculation purposes.

### Interferon-γ and interleukin-2 fluorospot assay

Antigen-specific interferon-γ (IFN-γ), interleukin 2 (IL-2), and double-positive IFN-γ^+^/IL-2^+^ cytokine-secreting T cells were quantified at the single-cell level with the FluoroSpot assay (Mabtech AB, Sweden). Briefly, 200.000 PBMCs/well were stimulated in duplicate with SARS-CoV-2 peptides (1 μg/mL), BPL inactivated SARS-CoV-2 (equivalent to moi = 1), negative controls (DMSO, medium alone) and anti-CD3 antibody (positive control). Plates were incubated for 16 hours overnight (37°C, 5% CO_2_) and developed according to the manufacturer’s instructions. The average spot forming units (SFU) of duplicates were counted using a fluorescence reader fitted with color filters for FITC and Cy3 (Advanced Imaging Devices, Germany) and background from negative controls were subtracted.

### Analysis

Data were calculated using Prism-v.8.4.2 (GraphPad). Demographic, clinical characteristics were examined using Chi-square and Fisher´s tests in SPSS (version 26). Multiple lin*ear regression analysis and adjusted ORs was calculated using a generalized linear regression model in R studio Version 1*.*2*.*5042*. Serological data were log-transformed and compared between time points. P*-*values <0.05 were considered statistically significant.

## Results

### Patient characteristics

Fifty-two patients (14 hospitalized and 38 community) were followed up with two- and six-months blood samples (Table 1 shows the demographics of the SARS-Cov-2 infected patients recruited during the first pandemic wave). The median time from diagnosis to follow up was similar in the two groups (182 vs 188 days) and the majority of patients were male (52 vs 57%). The age and gender distribution was similar in the two groups, however the community patients were younger, (mean 52 vs 60 years, although not significant) and had significantly less comorbidities (37% vs 79%) (p = 0.008) and lower BMI (median 24.5 vs 27.1 kg/m^2^) compared to hospitalized patients.

### SARS-CoV-2 specific antibody responses

**Fig 1:**
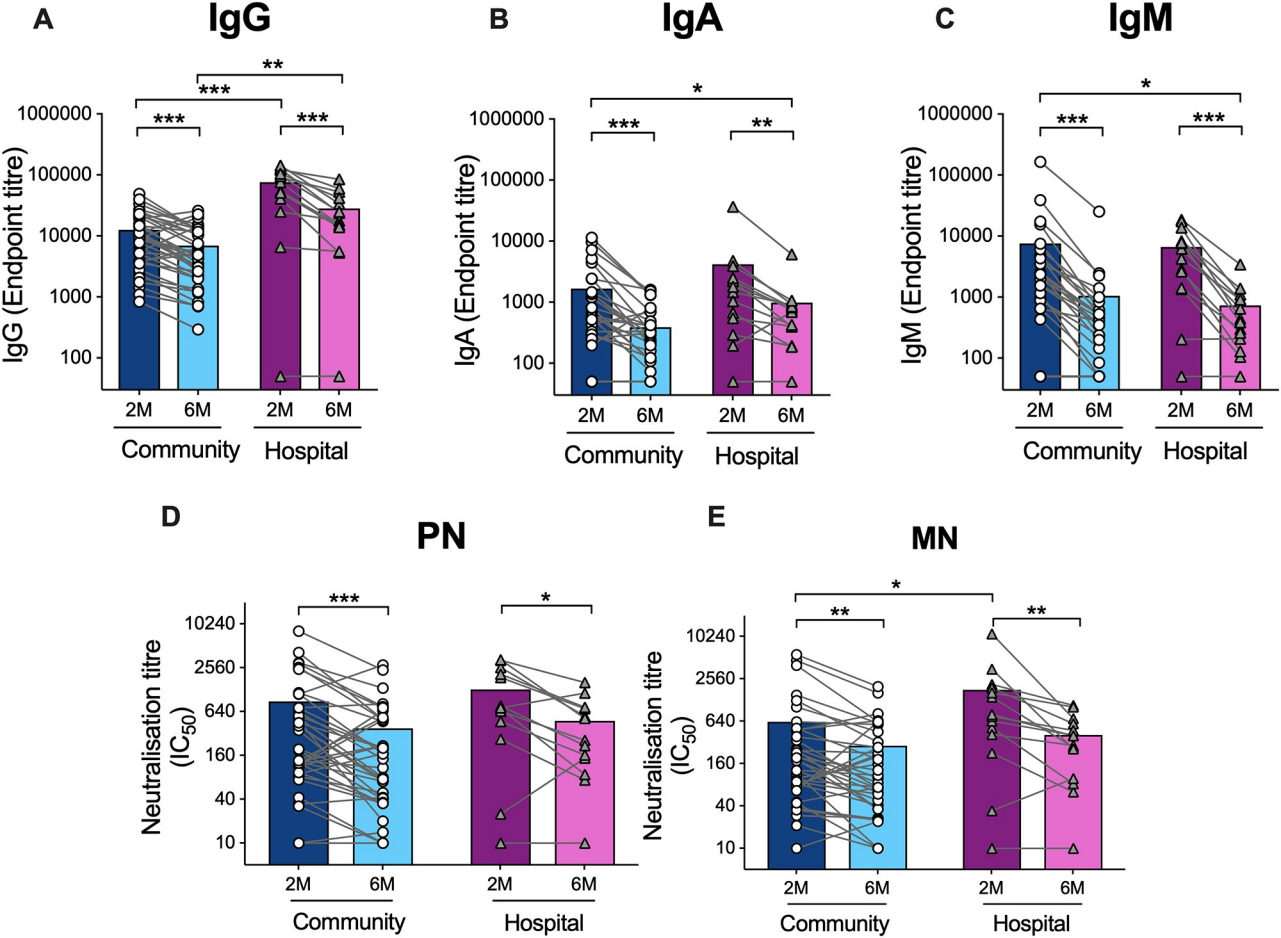
SARS CoV-2 specific antibody responses by severity of illness. Comparison of the SARS-CoV-2 spike-specific and neutralization antibody titers between community and hospitalized patients is shown, Spike-specific IgG(A), IgA (B), IgM (C). Serum was collected at 2- and 6-months post-infection, and spike-specific responses were measured by ELISA. The neutralization antibodies were measured by pseudotype neutralization (PN) (D) and microneutralization (MN) (E) assays. Each symbol represents the SARS-CoV-2 spike- specific antibody responses of one individual, and the lines connect the paired samples at 2 and 6 months. The bars represent the geometric mean titers. A nonparametric paired t-test (Kruskal–Wallis), was used to compare 2- and 6-month samples (* = P<0.05, **P<0.005).

SARS-CoV-2 specific antibodies were measured using a broad panel of assays to compare responses at 2- and 6-months post-infection. Hospitalized, severely ill patients had significantly higher spike-specific IgG compared to the outpatients at 2- and 6-months post-infection (Fig 1A, shows the SARS CoV-2 specific antibody responses by severity of illness).

At 2 months post-infection, hospitalized patients had significantly higher MN antibody titers, but not PN antibodies, while there were no significant differences at 6 months (Fig 1D and 1E show the comparison of SARS-CoV-2 pseudotype neutralization (PN) and microneutralization (MN) titers in community and hospitalized patients). A significant decline in IgG, IgA, IgM, PN and MN antibodies was observed from two to six months in both groups (community and hospitalized, respectively) (p = 0.004) (Fig 1A–1E shows the comparison of the SARS-CoV-2 spike-specific IgG, IgA, IgM, PN and MN antibody titers between community and hospitalized patients). Antibody levels waned by six months post-infection but remained above the cut-off level. All but one patient had spike specific IgG, and this individual did not mount an immune response in either the humoral or cellular immune compartment (Fig 1A shows the SARS CoV-2 specific antibody responses by severity of illness).

**Fig 2:**
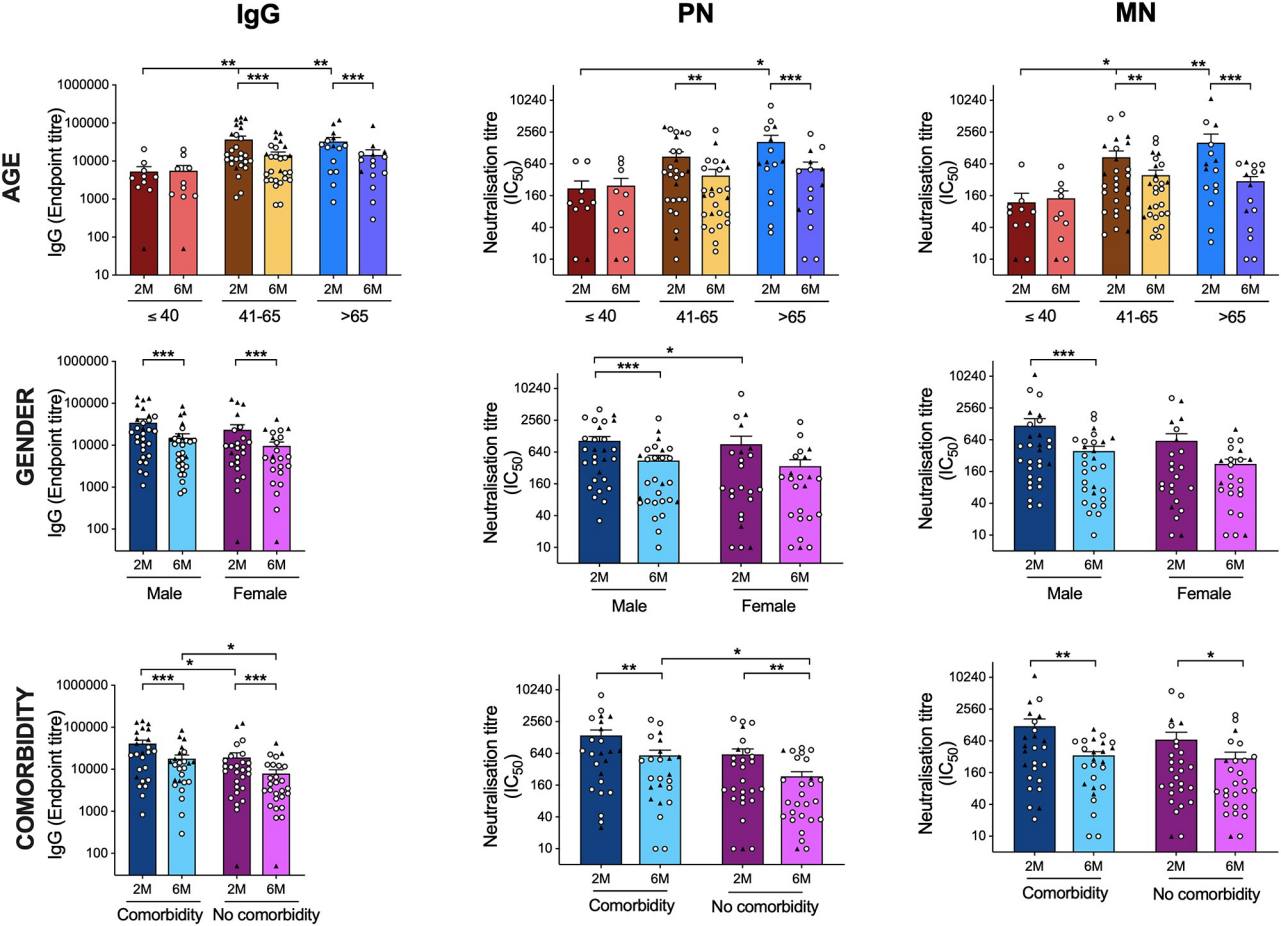
SARS CoV-2 antibody responses by age and gender. Comparison of the SARS-CoV-2 spike-specific and neutralization antibody titers according to age (A-C), gender (D-F) and the presence of comorbidities (G-I) is shown, Spike-specific IgG (A, D, G), PN (B, E, H) and MN (C, F, I). Each symbol represents the SARS-CoV-2 antibodies response from one individual with the circle symbol represents community dwelling patients, and the triangle represents hospitalized patients. The horizontal bars represent the mean T-cell response for each time point ± standard error of the mean. Statistical significance was determined by the non-parametric Kruskal-Wallis multiple comparisons test (* = P<0.05).

To reflect clinical observations, we analyzed antibody responses according to age, gender and presence of comorbidities. The IgG, PN and MN antibody levels were significantly highest in the oldest age group (>65 years old) at 2 months and waned significantly by 6-months (Fig 2A–2C, shows the SARS CoV-2 antibody responses by age). Globally, higher mortality rates have been reported in males compared to females. We analyzed antibody responses according to gender and found that 2 months post-infection, males had higher binding and neutralizing antibodies than females (Fig 2D–2F shows the comparison of the SARS-CoV-2 spike-specific and neutralization antibody titers according to gender) (IgG p = 0.06, MN p = 0.06, PN p = 0.03). At 6-months, both genders had similar levels of antibodies (Fig 2D–2F show the comparison of the SARS-CoV-2 spike-specific and neutralization antibody titers according to gender) (IgG, MN and PN p ≥0.16).

At 6 months patients with known comorbidities had higher spike-specific IgG and neutralizing antibodies PN (p<0.05), but not MN antibodies (p = 0.09) (Fig 2G–2I show the comparison of the SARS-CoV-2 spike-specific and neutralization antibody titers according to comorbidity). However, there was a strong correlation between PN and MN titers (S1 Fig shows the correlation between PN and MN antibody titers).

### SARS-CoV-2 specific T-cell responses, impact of severity, age, gender and comorbidities

**Fig 3:**
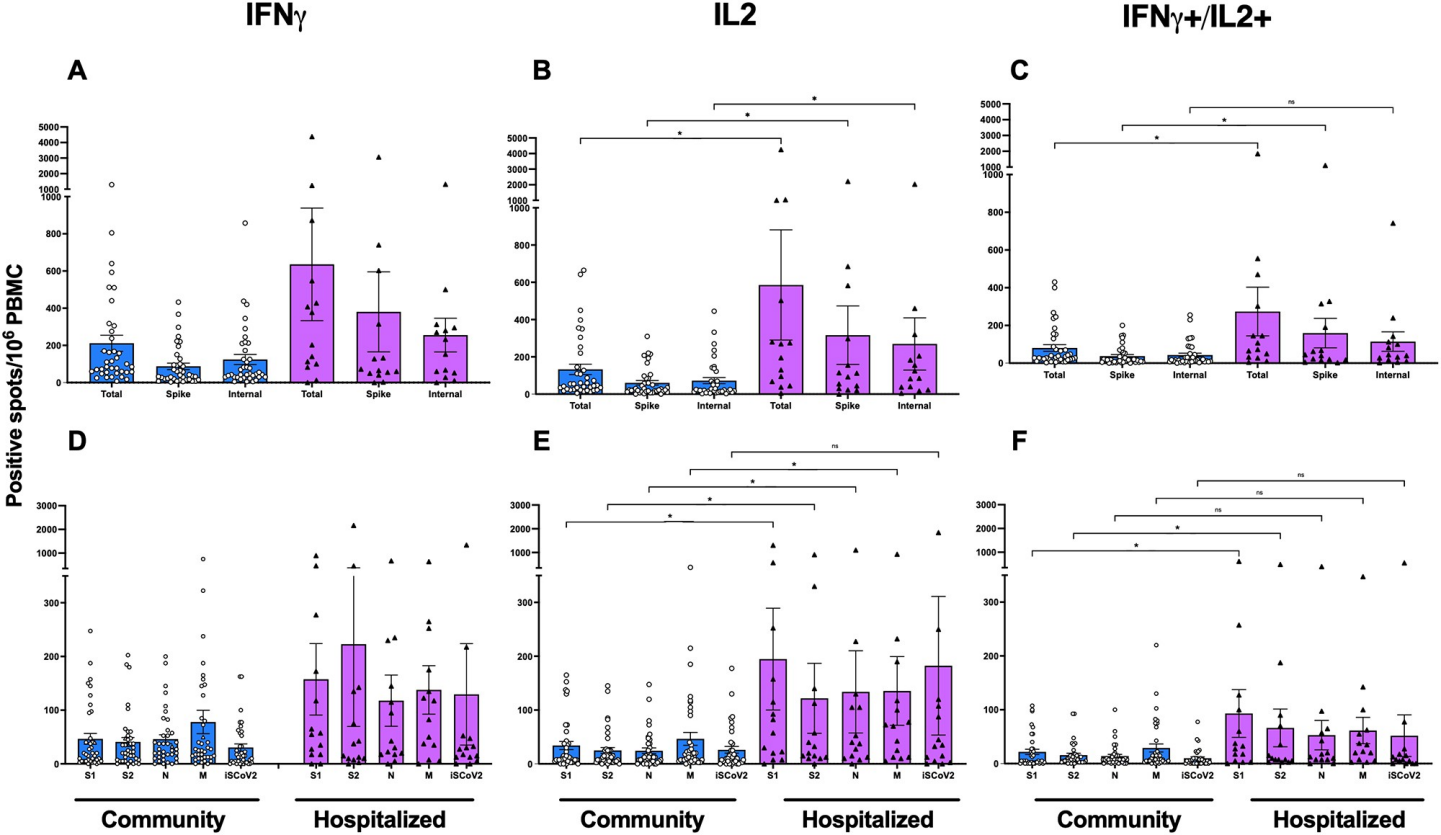
SARS CoV-2 specific T-cell responses in community and hospitalized patients. T-cell immune responses were evaluated by measuring the number of SARS-CoV-2 specific IFN-γ (A, D), IL-2 (B, E) and IFN-γ^+^+ IL-2^+^ (C, F) secreting T-cells, (spot forming units) (SFU) after infection using the FluroSPOT assay. A-C; peripheral blood mononuclear cells (PBMC) were stimulated with peptide pools to measure the total (S1, S2, N, M), internal (N and M), and spike (S1 and S2) specific SARS-CoV-2 responses. D-F; the SARS CoV-2 specific S1, S2, M, N and inactivated SARS CoV-2 hCoV-19/Norway/Bergen-01/2020 virus (ISCoV-2). Each symbol represents the SARS-CoV-2 IFN-γ/IL-2 response (spot forming units (SFU) per 1×10^6^ cells) after stimulation with virus spike antigen. The horizontal bars represent the mean IFN-γ response for each time point ± standard error of the mean. Statistical differences between different antigens or hospitalized and community dwelling subjects were determined by the nonparametric Kruskal–Wallis multiple comparisons test (* = P<0.05, ** = P<0.005).

To study differences in T-cell responses in the mildly (community) and severely ill (hospitalized) patient cohorts, we compared the SARS-CoV-2 specific IFN-γ, IL-2 and double positive (IFN-γ+/IL-2+) responses 6-months post-infection using specific peptide pools (Fig 3 shows the SARS CoV-2 specific T-cell responses in community and hospitalized patients). We observed a trend of higher IFN-γ spike (S1, S2) and internal (N, M) specific T cells in the hospitalized group compared to the community group (Fig 3A and 3D show the SARS CoV-2 specific IFN-γ specific T-cell responses in community and hospitalized patients). Significantly higher levels of IL-2 specific responses were found in the hospitalized compared to the community patients for the total (S1, S2, N, M), internal, and spike specific SARS-CoV-2 T cells (p<0.05) (Fig 3B and 3E show the SARS CoV-2 specific IL-2 specific T-cell responses in community and hospitalized patients). Similarly, the double positive responses were significantly higher in the total and spike specific but not internal antigens (p<0.05) (Fig 3C and 3F show the SARS CoV-2 specific IFN-γ+/IL-2+ specific T-cell responses in community and hospitalized patients).

**Fig 4:**
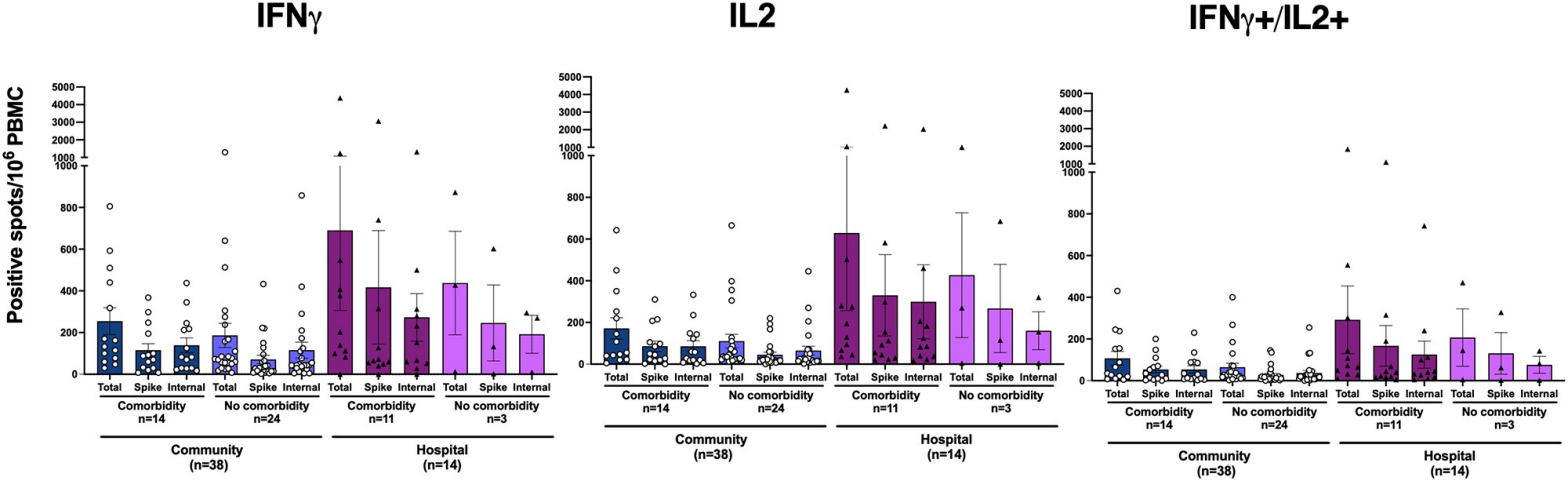
SARS CoV-2 specific T-cell responses by comorbidity. The SARS-CoV-2 specific T-cell responses in peripheral blood mononuclear cells (PBMC) were determined by IFN-γ^+^, IL-2^+^, and IFN-γ^+^+ IL-2^+^ in FluroSPOT in community and hospitalized patients who were SARS-CoV-2 confirmed, rt-PCR positive. The results are plotted according to the presence of comorbidities or no comorbidities. Each symbol represents the SARS-CoV-2 IFN-γ/IL-2 response (spot forming units (SFU) per 1×10^6^ cells) after stimulation with virus spike antigen. The circle symbol represents community dwelling patients, and the triangle represents hospitalized patients. The horizontal bars represent the mean T-cell response for each time point ± standard error of the mean. Statistical significance was tested by the non-parametric Kruskal-Wallis multiple comparisons test (P<0.05), and no significant difference was found.

**Fig 5:**
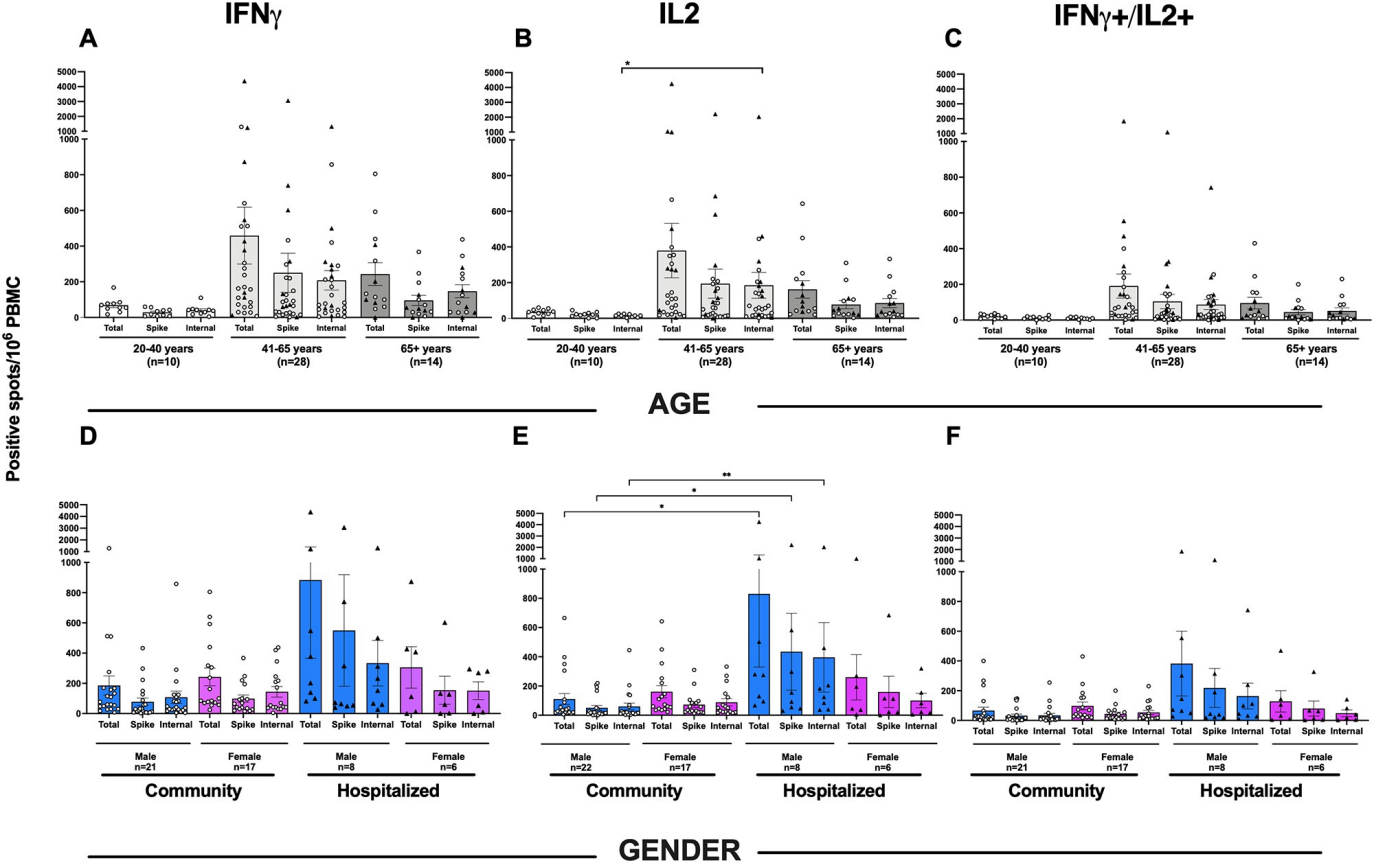
Influence of age and gender on SARS CoV-2 specific T cells. The SARS-CoV-2 specific IFN-γ responses in peripheral blood mononuclear cells (PBMC) were determined by IFN-γ^+^ (A,D), IL-2^+^ (B,E), and IFN-γ^+^+ IL-2^+^ (C,F), in FluroSPOT in community and hospitalized patients who were SARS-CoV-2 confirmed, rt-PCR positive. The results are plotted according to age (A-C) and gender (D-F). Each symbol represents the SARS-CoV-2 IFN-γ/IL-2 response (spot forming units (SFU) per 1×10^6^ cells) after stimulation with virus spike antigen. The circle symbol represents community dwelling patients, and the triangle represents the hospitalized patients. The horizontal bars represent the mean response for each time point ± standard error of the mean. Statistical significance was determined by the non-parametric Kruskal-Wallis multiple comparisons test (* = P<0.05, ** = P<0.005).

Comorbidities and age are risk factors for severe disease. By 6 months patients with comorbidities had higher frequencies of specific IFN-γ and IL-2 producing T cells; (spike and internal) although not significant when adjusted for severity of disease (hospitalization) (Fig 4A and 4B show SARS CoV-2 IFN-γ specific T-cell responses by comorbidity), When stratifying by age, the lowest antibody and T-cell responses were found in the youngest group (<40 years old), all of whom were community patients with less severe disease (Fig 5A–5C show the influence of age on SARS CoV-2 specific T cells). Interestingly we saw a trend of higher SARS-CoV-2 specific T cells in the middle age-group (41–65 years) (Fig 5A shows the influence of age on SARS CoV-2 specific T cells), followed by the elderly (65+ years), although only statistically significant (p<0.05) for the IL-2 towards the internal peptides (N, M) (Fig 5B shows the influence of age on SARS CoV-2 specific T cells). We did not find differences in T cell responses according to gender and the gender distribution was equal in the hospitalized and community cohorts. However, the levels of IFN-γ producing T cells at 6 months were higher in males hospitalized compared to the community cohort, although this was only significant (p<0.05) for IL-2 producing T cells reactive against spike and internal peptides (Fig 5D–5F show the influence of gender on SARS CoV-2 specific T cells).

## Discussion

Current lack of specific treatment, correlates of protection and large variations in the clinical disease, underscores the need for detailed knowledge of human immune responses to SARS-CoV-2 [24]. Here, we conducted a prospective follow-up study of the first community- and hospitalized patients infected in Bergen, Norway to determine the long-term immune responses. We found durable cellular and humoral responses six months after rt-PCR-confirmed infection in patients with diverse illness severities. Although the protective effect of the sustained cellular immunity is unknown, the findings may have an impact on re-infection rates, vaccination strategies and infection control measures.

Virus-specific antibodies may inhibit de-novo infection, however cellular responses are crucial for activating, modulating, and maintaining B-cell responses, vital for the long-term protection against re-infection. T cells are involved in clearance of infected cells and tissue repair and can limit disease severity [25]. Our important findings of durable specific T cells and antibody responses in patients with both mild-to-moderate and severe disease are encouraging. These T cells may reduce the risk of re-infection with variants of concern, as killing of infected cells, reduction in viral load and transmission has been shown in animal models [15]. Furthermore, our findings are supported by reports from Denmark where mild and severe cases mounted either a humoral or cellular response, and from the original outbreak city of Wuhan; with the majority of immune-responses lasting nine months post-infection [26–28]. Specific T cells were also found in patients with only mild/asymptomatic disease and their antibody negative household members [16]. Interestingly, studies after infection with SARS in 2003, provide support for durable immunity (>6 years) [17], as well as cross-reactive responses to SARS-CoV-2 17 years later [29]. Although the extent to which this cross-reactive T-cell memory can protect against SARS-CoV-2 is not known.

Although most subjects mounted a humoral or cellular response, we found a lack of immune response in some individuals, similarly to Nielsen and coworkers [26], which could increase the risk of re-infection with variants of concern. Regardless of severity, and without known immunological deficiencies, one hospitalized patient did not mount an antibody nor a cellular response, providing a possible immunological explanation for the observation of cases of re-infection.

Patients with comorbidities are at higher risk of developing severe COVID-19 than healthy subjects, and Covid-19 disease is dependent on the host immune response to infection, hence we aimed to analyze the immune responses according to known risk factors for severe disease. Our SARS-CoV-2 specific serological and cellular findings show significantly higher titers in those with severe disease, and an association with comorbidity, gender and age although not significant. Our findings are supported by another study which found that male gender, older age, and hospitalization for COVID-19 were associated with increased antibody responses [30]. The lowest T cells responses were found in the youngest age-group (20–40 years), perhaps due to their mild disease since all were community patients, as adaptive immune responses post-SARS-CoV-2 infection are associated with age and severe disease [25]. In support of our findings, an Indian study found persistent T-cell responses in mild cases [31]. Our findings of significantly higher IL-2^+^ T-cell responses in the middle-aged group could be linked to a cross-reactive memory response to conserved epitopes in human coronaviruses (HCoV), while the decrease in the oldest group could be linked to immunosenescence. Indeed 20–28% of healthy controls who had no infection with SARS-CoV-2, had low levels of specific T cells, suggesting some cross reactivity to HCoVs [16, 32]. Furthermore, our results are supported by the observation of a negative correlation between low T-cell responses and age [5].

Males often suffer from more severe COVID-19 illness, with lower T cell responses reported to be associated with more severe disease in males compared to females [5]. We did not find significant differences in T-cell responses between genders or between those with or without comorbidities when adjusting for severity of disease, although there was a trend of higher responses in men. This could be due to a low number of subjects. Hospitalized men had significantly higher T cell responses (IL-2) compared to community dwelling men (Fig 5E shows the influence of gender on SARS CoV-2 specific T cells), indicating that severity of disease is related to increased cellular responses.

Obesity has also been associated with a higher risk of severe disease [33]. Overall, the mean BMI was lower in the community than hospitalized patients (24 vs 27 kg/m^2^ respectively), and lower compared to global reports [34, 35].

Neutralizing antibodies to the spike protein and its receptor binding domain (RBD) of SARS-CoV-2 prevent the virus binding to epithelial cells in the upper airways through its receptor angiotensin-converting enzyme 2 (ACE2), providing protective immunity after infection or vaccination. The waning of antibody responses over time, combined with emerging variant viruses with increased transmissibility, have increased the chances that SARS-CoV-2 will continue to circulate, perhaps becoming a regularly circulating seasonal virus. Significantly lower IgG and IgM were found in asymptomatic cases who tested positive for SARS-CoV-2, than in symptomatic patients [36]. The patients in our study, were all symptomatic and had sustained and significantly higher spike-specific antibodies (IgG, IgA, IgM, MN, but not PN), with the highest levels in those hospitalized, perhaps due to a higher initial viral load eliciting a strong initial immune response [37]. SARS-CoV-2 serology has been found to be more sensitive than rt-PCR for detecting people who have undergone infection [18, 38].

Age is the most important risk factor for severe disease and mortality from SARS-CoV-2 infection and may explain our findings of significantly higher antibody titers in patients with severe disease and with increasing age, in agreement with other studies [39, 40]. Although there is no agreed correlate of protection, high levels of specific antibodies appear to be a biomarker for severe disease [19, 41]. Moreover, variations in laboratory methodology globally may make this even more complex to stratify, but the use of WHO international antibody standards will allow global comparison of antibody titers [42].

Interestingly, all infected subjects in our study had detectable neutralizing SARS-CoV-2 antibodies, with only one non-responder. The evidence of the vital role of antibodies in preventing re-infection was documented during an outbreak aboard a ship [43], where neutralizing antibodies from prior infection was significantly associated with protection against re-infection. However, the immune response during the acute phase or in the early convalescent phase after recovery, did not predict the long-term protective immune response [39]. Re-infection has occurred in previously infected people, indicating that durable protection may not be achieved in all individuals [44, 45]. Furthermore, studies of the durability and breadth of neutralizing antibodies are needed to understand if there is a role for herd immunity in preventing long term complications after SARS-CoV-2 infection [19].

A key question in understanding the clinical course of SARS-CoV-2 disease is how the initial antibody response determines the course of the primary illness, and to what extent the serological response impacts long-term complications. Antibody titers during initial illness have been found to correlate with symptoms of post-acute COVID-19 syndrom or “long-covid” 6 months post-infection, even in mild disease [19]. Most patients seroconvert within a week of SARS-CoV-2 infection, however IgG has been found to persist up to 8 months after infection, while local IgA and IgM declined more rapidly [8, 28, 46–48]. Importantly, we found durable antibody responses in some patients with undetectable cellular responses, supporting the complexity of the immune response to SARS-CoV-2 with the different immune compartments responding individually [28]. Recent data on spike protein-based vaccines highlights the importance of these spike specific antibodies in protection from disease, hospitalizations and deaths.

Our findings of post-infection T cells binding to more conserved internal viral epitopes, provides the possibility of cross-reactive T cell protection, as has been observed after the 2009 H1N1 influenza pandemic and the avian H7N9 avian flu outbreak [49, 50]. Spike-specific T cells have been found in SARS-CoV-2 patients, including in mild disease, and 30–50% of healthy people without infection were found to have SARS-Cov2 specific CD4+ and CD8+ cytotoxic T cells [31, 51, 52], possibly HCoV cross-reactive T cells. Moreover, the observation of children having less severe COVID-19 disease may be due to cross-reactive T cells from multiple earlier infections with seasonal HCoVs [53, 54].

Although limited by small numbers of patients, the durable antibody and cellular responses found in our study may provide protection against re-infection or be boosted after vaccination and is supported by a US study [28]. However, the level of cross-reactivity is unknown, especially in patients with mild disease. Encouraging, in patients surviving severe disease, heterogenous long-term T-cell responses have been found up to 9 months post-infection from patients residing in Wuhan [27, 28]. The advantages of our prospective cohort study are the early recruitment of both hospitalized and community patients during the first pandemic wave and the broad investigation of immune responses, including two methodologies for neutralizing antibodies to assess potential protection.

## Conclusion

We found the highest cellular immune responses in the middle-aged and in hospitalized patients with comorbidities, reflecting clinical observations that older age, and comorbidities are related to severe disease. Durable T-cellular immune responses in community patients, with mild disease, suggests that patients surviving SARS-CoV-2 infection may be partially protected from re-infection with variants of concern. Upon re-exposure or vaccination, long-term immunity will be boosted. Such boosting may possibly provide protection although the level of cross-reactivity to variants of concern needs to be determined.

## Supporting information

S1 Fig Correlation between PN and MN antibody titers. The figure shows the correlation of the SARS-CoV-2 spike-specific micro neutralization antibody titers (MN) and virus peudotype neutraliazation titers (PN) at 2 months post-infection (A) and 6 months post-infection (B) in community and hospitalized patients. Each symbol represents the SARS-CoV-2 PN or MN antibody response from one individual with the circle symbol representing community dwelling patients, and the triangle representing hospitalized patients. The horizontal bars represent the mean T-cell response for each time point ± standard error of the mean. Statistical significance was determined by Spearman correlation test (A): r = 0,914, p = 0^e^ +000 and (B) r = 0,924, p = 0^e^ +000.
(TIFF)

S1 Data (XLSX)
