# Supplementary figures and images for: Durable T-cellular and humoral responses in SARS-CoV-2 hospitalized and community patients
Source: PLoS One. 2022 Feb 22;17(2):e0261979. doi: 10.1371/journal.pone.0261979 (PMC8863217; doi:10.1371/journal.pone.0261979)

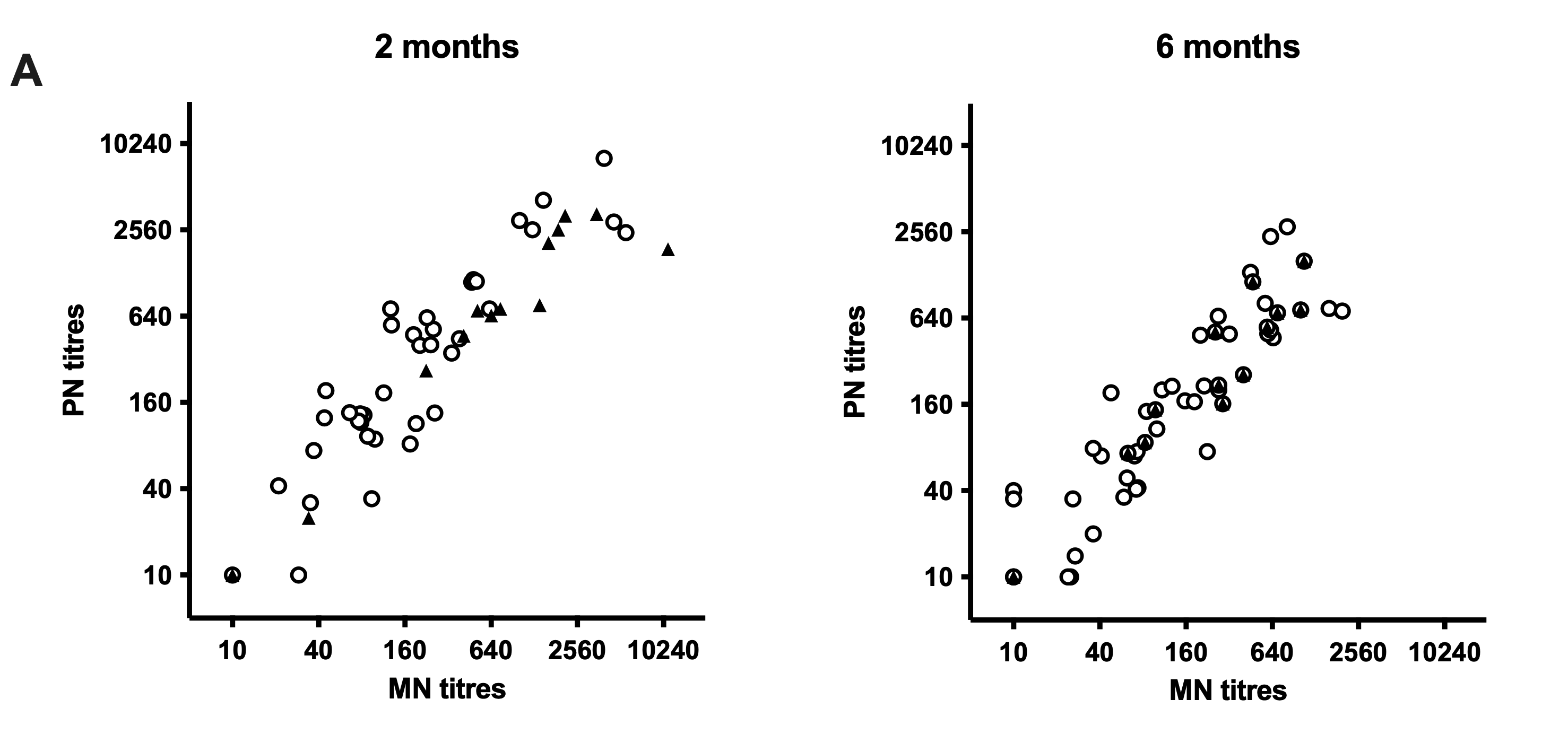

Supplement: S1 Fig — The figure shows the correlation of the SARS-CoV-2 spike-specific micro neutralization antibody titers (MN) and virus peudotype neutraliazation titers (PN) at 2 months post-infection (A) and 6 months post-infection (B) in community and hospitalized patients. Each symbol represents the SARS-CoV-2 PN or MN antibody response from one individual with the circle symbol representing community dwelling patients, and the triangle representing hospitalized patients. The horizontal bars represent the mean T-cell response for each time point ± standard error of the mean. Statistical significance was determined by Spearman correlation test (A): r = 0,914, p = 0e +000 and (B) r = 0,924, p = 0e +000. (TIFF) [file pone.0261979.s001.tiff]
